# Asymmetric Partisan Voter Turnout Games

**DOI:** 10.1007/s13235-021-00384-1

**Published:** 2021-04-16

**Authors:** Cameron Guage, Feng Fu

**Affiliations:** Dartmouth College, Hanover, NH 03755 USA

**Keywords:** Downs’ paradox, Evolutionary game dynamics, Evolutionarily stable strategies, Social learning, Asymmetry

## Abstract

Since Downs proposed that the act of voting is irrational in 1957, myriad models have been proposed to explain voting and account for observed turnout patterns. We propose a model in which partisans consider both the instrumental and expressive benefits of their vote when deciding whether or not to abstain in an election, introducing an asymmetry that most other models do not consider. Allowing learning processes within our electorate, we analyze what evolutionarily stable strategies are rationalizable under various conditions. Upon varying electorate size, the partisan split of the electorate, and the degree to which an electorate takes underdog considerations into account in its payoff structure, we find that different equilibria arise. Our model predicts comparative statics that are consistent with voter behavior, specifically affirming a “size effect,” in which turnout decreases as electorate size increases. Furthermore, relaxing some of our preliminary assumptions eliminates some of the discrepancies between the predictions of our model and empirical voter behavior. In particular, our work demonstrates that misperceptions about the partisan split of an electorate may account for high turnout behavior .

**Supplementary Information:**

The online version supplementary material available at 10.1007/s13235-021-00384-1.

## State of the Art

A problem that has plagued political scientists for decades without satisfactory resolution is the apparent irrationality of voting. One should only vote if the benefits outweigh the costs, and, if voting is considered to be an instrumental means to an end of effecting political change, this is rarely the case [11]. In the discussion of this anomaly, the payoff an individual receives for voting is typically represented by the following expression:

$$\begin{aligned} \mathrm{pB}-{ c} \end{aligned}$$

where *p* is the probability that one’s vote will be pivotal, *B* is the benefit differential one derives from one’s preferred candidate gaining office, and *c* is all opportunity costs associated with the act of voting.

Individuals only choose to vote if this expression is positive, an outcome that is extremely unlikely when considering the remarkably small values of *p* inherent in most elections. For an American presidential election, this value is less than one in ten million [41]. Furthermore, in an empirical study on US Congressional and state elections, Mulligan and Hunter [28] find that only one out of every 100,000 votes cast in US elections was cast for a candidate that either tied or won by a single vote. The fact that *p* is likely to be so minuscule, then, should deter any individual with even the most negligible voting costs from going to the polls, driving turnout rates effectively to zero. Paradoxically, a wise individual would realize that, with no other rational individuals voting, *p* would effectively become one, and she (along with all other individuals) should vote, again decreasing *p*, and so on.

The seeming irrationality of one of the most fundamental acts of democracy set many political scientists and economists into motion in an attempt to explain how rational individuals can vote with high turnout rates, even when facing non-negligible costs. Initially, Riker and Ordeshook [34] offered the potential solution that Downs’s model was incomplete, and that there should be another benefit term that is not scaled by the probability that one’s vote is pivotal. This term represents a consumption benefit one derives from voting, and could be seen as the satisfaction one receives from fulfilling one’s “civic duty” via electoral participation. They explain that adding a variable (they use *D* in their payoff equation) for the consumption benefit one derives from voting accounts for the rationality of high voter turnout when it is clear that the probability that an individual’s vote will impact the outcome of the election is staggeringly small. However, many criticized this model for offering little insight into voting motivations, as the magnitude of one’s *D* term seems to almost entirely dictate the decision of whether or not to vote. Additionally, it does not account for behavior like changes in turnout in the same region for different types of elections [17]. Lastly, while the results from this model compare well with election data within a given year, they do not seem to hold up when analyzing election data across different years [1].

Another proposed solution to Downs’ paradox is that, rather than utility maximizers, rational individuals are regret minimaxers; that is, they choose the strategy that minimizes the chance of ending up with the result that would produce their maximum regret [15]. While supporting high turnout equilibria, the minimax regret model has been largely discounted in the literature, with critics pointing out that a true regret minimaxer would be so risk-averse that he would never cross the street, even if the polling location were on the other side [10].

Others have used a game-theoretic approach to help explain the phenomenon of voting. Ledyard [22] posits a model of voting behavior that includes voters and candidates as players in a voting game, but finds zero turnout in equilibrium. One of the more promising attempts to break the paradox of not voting was undertaken by Palfrey and Rosenthal [31]. They find that, in their model, high turnout behavior can be supported, even in the presence of high costs. Two years later, they build on this model by introducing uncertainty about voting costs, noting that the equilibria found in their 1983 paper were fragile insofar as they rested on the assumption that costs were common knowledge to the entire electorate. They find that, once some information about voting costs is restricted to individuals themselves, the high turnout equilibria vanish [33].

Others have attempted to use information in a different context to explain voting, referring to the information one has about the candidates and the potential political consequences of their elections to office. In an attempt to explain voting patterns,  Matsusaka [25] claims that the more information a citizen has, the higher the payoff she receives from voting is, as she is more confident in her vote. Similarly believing in the power of information to explain voter turnout,  Feddersen and Pesendorfer [13] create a model that assumes asymmetric information in a population; however, it is in discord with both Matsusaka’s formulation of the information effect on turnout and intuition, as abstention rates are at times positively correlated with the proportion of informed voters in the population.

A different voting model that has gained traction in the political science community as a potential solution to the paradox of not voting assumes that, rather than being entirely self-interested when deciding whether or not to vote, individuals are rule utilitarians [19]. Rule utilitarians follow a rule (for voting, in this setting) that, if followed by everybody, leads to the result that yields the maximum utility. Harsanyi does not indicate how this rule applies to situations in which there are divergent opinions regarding what the most socially desirable outcome is, which is undoubtedly the case when it comes to partisan politics. Feddersen and Sandroni [14] consider this possibility and allow disagreement about what the utility-maximizing outcome is, outlining a model where turnout is motivated by disagreement within an electorate. This type of model is corroborated by empirical evidence, with the group rule-utilitarian model explaining nearly half of the variation in voter turnout in Texas liquor referenda [9]. Contrary to this support, Merlo and Palfrey [26] find that, upon comparing this “ethical” voting model with many others using the concealed parameter recovery method, the ethical model performs relatively poorly. Furthermore, Feddersen [12] claims that group-based models such as the rule-utilitarian and altruistic voting model [24] are problematic as they do not ensure the existence of equilibria, allow for mixed strategy equilibria, or explain why people join groups in the first place, which he believes is relevant in the calculus of voting for group-based models.

Others have attempted to model voters as learners in a survival approach rather than rational utility maximizers. Sieg and Schulz [39] assume voters repeat strategies that induced pleasure and avoid strategies that induced punishment. They find that, while in some scenarios their model predicts Downs’ troubling result of zero turnout, it can also lead to individuals “learning” participation. Palfrey and Rosenthal [32] support the promising nature of this work, asserting that learning over time is a helpful tool for narrowing down the multiplicity of equilibria that may arise in voting games. Furthermore, evolutionary processes in electoral settings are supported by empirical studies such as the work of Rosenthal and Sen [36], who find that learning processes are present within the electorate of the French Fifth Republic. While Sieg and Schulz’s model certainly sheds light on turnout behavior, it does not allow for any mixed equilibria, a possibility that is worth considering when we observe similar people behaving in divergent ways in electoral settings.

Many others have conducted experiments and empirical studies to examine voter behavior and test the validity of some of the different voter models proposed in the literature. Some have studied the effect of different representation systems on turnout [3, 37], while others have focused on the comparative statics of election data to observe how different electorate characteristics relate to turnout. Many studies have found a “size effect,” wherein turnout decreases as electorate size increases [2, 8, 18, 23, 35], and many have found a “closeness effect,” wherein turnout decreases as the closeness of an election decreases [23, 40], although closeness can be evaluated in different ways. Kaniovski and Mueller [21] try to explain these relationships qualitatively, claiming that electoral closeness and size reflect heterogeneity in an electorate, which in turn increases voter turnout. Other studies use these results as evidence that voters are rational actors that follow Downs’ voting utility formulation, as their votes are more likely to be pivotal in small electorates and close elections. The consensus seems to be that, while this evidence supports the idea that voting is not solely motivated by a “consumption benefit” that one invariably gets from going to the polls, it is not solely motivated by pivotal-vote considerations either. For example, Breux, Couture, and Goodman [8] find that rational choice theory can explain approximately 45% of voter turnout in municipal elections. An empirical study of presidential elections found that, while the rational voter hypothesis seems unable to explain turnout in its entirety, neither can “civic duty” arguments [16]. Green and Shapiro [17] also note that rational utility maximization is just a part of the explanation for why people vote.

In fact, there is a growing literature against homo economicus, or the idea that human behavior, and in this case voting behavior, should be viewed strictly through the economic lens of utility maximization. This is not to say that voters are irrational, but rather that they have payoff structures that reflect considerations Downs did not include. One can then analyze how rational individuals would act in this new voting setting that is more reflective of political considerations. Overbye [30] challenges homo economicus by blending economic and sociological concepts when analyzing voter behavior. He conceptualizes voting as an investment in the reputation that one is concerned about the public good. Brennan and Lomasky [7] point out that political and economic behavior are inherently different, with political behavior having not only the instrumental benefit that an economic decision has, but also an expressive benefit. Schuessler [38] describes this expressiveness as a benefit associated with “Being,” as opposed to the instrumental benefit associated with “Doing.” Morgan and Várdy [27] examine the interplay between instrumental and expressive benefits in the context of a Condorcet jury model in order to examine optimal electorate size. Borah [5] considers the interplay between instrumental and expressive motivations in a more general context, finding that expressive concerns dominate instrumental concerns in larger electorates, a finding consistent with the work of Morgan and Várdy [27]. Brennan [6] explains that humans exhibit expressive behavior all the time, from going to watch one’s favorite team play football to sending a get well soon card to a relative. Importantly, he notes that expressive behavior is not necessarily outcome-independent, which would have an effect similar to that of the Riker–Ordeshook model’s *D* term. With this idea in mind, we posit a game-theoretic model for voting behavior that blends instrumental and expressive motivations in an attempt to better understand the dynamics behind voter turnout in partisan elections. We outline our base model in Sect. 2. In Sect. 3, we analyze this model and its implications for voter behavior. Section 4 compares the turnout predictions of our model with trends in real electoral data. Section 5 discusses some possible extensions to our model, and in Sect. 6, we conclude.

## Base Model

Our model assumes a two-candidate election in which each member of the electorate has preferences regarding which candidate’s election to office they perceive will lead to a superior outcome. While many countries, especially those in Europe, have elections that are contested by three or more parties, we analyze the two-candidate scenario here for both relevance to the context of the USA and simplicity (although applying this model to three or more candidates would be a fruitful topic for future research). The electorate can then be partitioned into two blocs: one that supports the first candidate, which we will henceforth call candidate *A*, and one that supports the second candidate, which we will refer to as candidate *B*. We further assume that individuals will not vote for the candidate they do not support, a finding consistent with the work of Herzberg and Wilson [20], who claim that citizens tend to vote sincerely. For simplicity, we assume that more people support candidate *A* than candidate *B*. We consider an *N* person election in which $p_\mathrm{A}$ (>.5) is the proportion of the electorate that supports candidate *A* and $p_\mathrm{B}$ $(= 1 -p_\mathrm{A}$) is the proportion of the electorate that supports candidate *B*. We assume a simultaneous-vote and winner-take-all (majority-rule) election, lest pivotal concerns become irrelevant, as they could in a system of proportional representation. Each individual in our model then faces a binary decision: vote for her candidate of choice, or abstain. Furthermore, there are three distinct electoral outcomes: one’s candidate of choice wins, one’s candidate of choice loses, or one’s candidate of choice ties. With this in mind, we define the payoff structure for a supporter of candidate $j \in {(A, B)}$ as follows:

$$\begin{aligned} {\left\{ \begin{array}{ll} -1 &{} \text {Vote and candidate } j \text { loses} \\ \ 1-c(p_j - .5) &{} \text {Vote and candidate } j \text { wins} \\ \ 1/2 &{} \text {Vote and candidate } j \text { ties} \\ \ 0 &{} \text {Abstain} \\ \end{array}\right. } \end{aligned}$$

To provide rationale for these payoffs, we urge the reader to participate in a thought experiment. In a similar vein to the work of Brennan [6], our quasi-expressive voting model can be compared to the expressive act of seeing one’s favorite football team play. When considering whether or not to go to the game, one weighs the potential benefits with the costs of attendance, such as gas, tickets, and the opportunity cost of foregone leisure. If one’s preferred team is going to lose, one is better off staying at home and not incurring the costs of attendance. That is, there is no expressive benefit to attending the game if one’s team of choice loses; nobody wants to walk out of the stadium wearing the colors of the losing team, and one could have done better by staying home.

However, if one’s team is going to win, one will usually want to attend the game. It is not enough to just be a supporter of the winning team; if one wants to reap the benefits associated with victory, be it the high fives with other fans or storming the field upon victory, one must have gone to the game. In this case, one is glad to have expressed one’s preferences, even with the knowledge that one’s cheering likely did not affect the outcome of the game. Thus, there is an expressive benefit for showing up to contribute to one’s favorite team when this team wins.

Furthermore, we believe that the utility one gets from attending a game in which one’s team is victorious is affected by how much one was expecting one’s team to win. One can imagine a game in which team *A* is almost sure to beat team *B*. The joy imparted to an *A* fan when they nearly inevitably win would certainly be different than the joy imparted to a *B* fan who risked coming to the game and saw a shocking underdog victory over the mighty team *A*. Thus, our model includes what we call an “underdog consideration,” wherein the majority’s winning payoff is decreasing in the magnitude of the majority, and the minority’s winning payoff is increasing in the magnitude of the majority. In our case, $p_j$ is a proxy for individuals’ perceptions about the likelihood that candidate *j* will win the election. The *c* variable is a non-negative coefficient (an assumption we will relax later) that scales the degree to which a particular electorate factors this underdog consideration into its payoff structure.

If one’s favorite team ties, the game goes into overtime; that is, a sure loss is prevented. In electoral politics, there is a surprising lack of consistency regarding the resolution of ties, with tiebreakers like coin flips, re-elections, and, in the 2020 Iowa caucus, even card draws deciding the fates of the candidates. Regardless, a sure loss is almost always prevented when an electoral tie occurs. It is noteworthy that, in our payoff structure, the payoff for voting when the election results in a tie is always positive, and in fact can exceed the payoff for voting when one’s candidate of choice wins. In our football analogy, one might imagine that, however unlikely, one’s presence at a game that ends in a tie may have played a role in preventing one’s favorite team from losing the game: that if one less voice had been cheering, the team might just have lost the game (from a lack of motivation or something of the sort). One’s role in propping up this victory is what motivates this payoff. If this seems far-fetched, consider the electoral context. In the case of a tie, it is not an exaggeration to think that one’s vote prevented one’s candidate of choice from losing the election; in fact, one would err in saying otherwise. Not only is the individual we are considering pivotal in preventing a loss, but also so is every individual who voted for the same candidate, as well as all of the individuals who voted for the other candidate, resulting in no negative payoffs in the electorate if this is the electoral outcome. While voting is certainly an expressive action, it would be ignorant to assume that individuals do not consider the instrumental impact of their vote, and that they would not derive benefit from playing a pivotal role in preventing the electoral defeat of their preferred candidate.

Now, when it is time to decide whether or not to go to the football game, the fans of each team (or, as one can likely infer by now, the supporters of each party) now face a decision: given the possible outcomes and one’s respective preferences over those outcomes, is leaving the costless but benefit-less comfort of home worthwhile?

If we weigh the potential payoffs for voting by their respective likelihoods of occurring and sum over the three possible cases, we can obtain the expected payoff for voting. If $V_j$ is the number of individuals that vote for candidate *j*, then the payoff an *A* supporter expects to receive for voting is

$$\begin{aligned} P(V_\mathrm{A} > V_\mathrm{B})(1-c(p_\mathrm{A}-.5)) + P(V_\mathrm{A} < V_\mathrm{B})(-1) + P(V_\mathrm{A} = V_\mathrm{B})(1/2) \end{aligned}$$

And the payoff a *B* supporter expects to receive for voting is

$$\begin{aligned} P(V_\mathrm{A} > V_\mathrm{B})(-1) + P(V_\mathrm{A} < V_\mathrm{B})(1-c(p_\mathrm{B}-.5)) + P(V_\mathrm{A} = V_\mathrm{B})(1/2) \end{aligned}$$

Comparing this expected payoff to the sure zero payoff for abstaining, individuals will choose to vote when the expected payoff for voting is greater than zero and abstain when it is less than zero.

As all citizens face the binary choice of vote versus abstain, we can model the number of individuals who turn out to vote for each party as a binomial random variable. Thus, while we assume symmetry within parties, there is an asymmetric aspect to our game, insofar as voters from different parties can decide to vote with different probabilities. If supporters of candidate *j* will vote with probability $q_j$, then $V_\mathrm{A} \sim \mathrm{binom}(\mathrm{Np}_\mathrm{A}, q_\mathrm{A})$, and $V_\mathrm{B} \sim \mathrm{binom}(\mathrm{Np}_\mathrm{B}, q_\mathrm{B})$.

As the probability that candidate *A* wins the election is $P(V_\mathrm{A} > V_\mathrm{B})$, this can be expanded to

$$\begin{aligned} \sum _{k=0}^{Np_\mathrm{B}}\left( {\begin{array}{c}Np_\mathrm{B}\\ k\end{array}}\right) (q_\mathrm{B})^k(1-q_\mathrm{B})^{Np_\mathrm{B}-k} \sum _{j=k+1}^{Np_\mathrm{A}}\left( {\begin{array}{c}Np_\mathrm{A}\\ j\end{array}}\right) (q_\mathrm{A})^j(1-q_\mathrm{A})^{Np_A-j} \end{aligned}$$

Similarly, the probability that candidate *B* wins is

$$\begin{aligned} \sum _{k=0}^{Np_B-1}\left( {\begin{array}{c}Np_\mathrm{A}\\ k\end{array}}\right) (q_\mathrm{A})^k(1-q_\mathrm{A})^{Np_\mathrm{A}-k} \sum _{j=k+1}^{Np_B}\left( {\begin{array}{c}Np_\mathrm{B}\\ j\end{array}}\right) (q_\mathrm{B})^j(1-q_\mathrm{B})^{Np_\mathrm{B}-j} \end{aligned}$$

And the probability of a tie is

$$\begin{aligned} \sum _{k=0}^{Np_\mathrm{B}}\left( {\begin{array}{c}Np_\mathrm{A}\\ k\end{array}}\right) (q_\mathrm{A})^k(1-q_\mathrm{A})^{Np_\mathrm{A}-k}\left( {\begin{array}{c}Np_\mathrm{B}\\ k\end{array}}\right) (q_\mathrm{B})^k(1-q_\mathrm{B})^{Np_\mathrm{B}-k} \end{aligned}$$

In order to analyze equilibrium behavior in our model, we plot the points at which *A* and *B* supporters are indifferent between voting and abstaining (that is, when their expected payoffs for voting are exactly equal to zero) over $q_\mathrm{A}$ and $q_\mathrm{B}$ to see what turnout states are rationalizable.

We find that, upon varying our three main parameters (*N*, $p_\mathrm{A}$, and *c*), these indifference functions can be configured in different ways that have different implications for voter behavior. We go on to analyze the effects of changing these variables on voter behavior in the next section. While the formulation of our utility function takes on strong assumptions that drive our results, we believe it is worthwhile to explore what comparative static predictions this particular payoff structure would give rise to. We then go on to relax some of these assumptions in a later section.

## Analysis

**Fig. 1:**
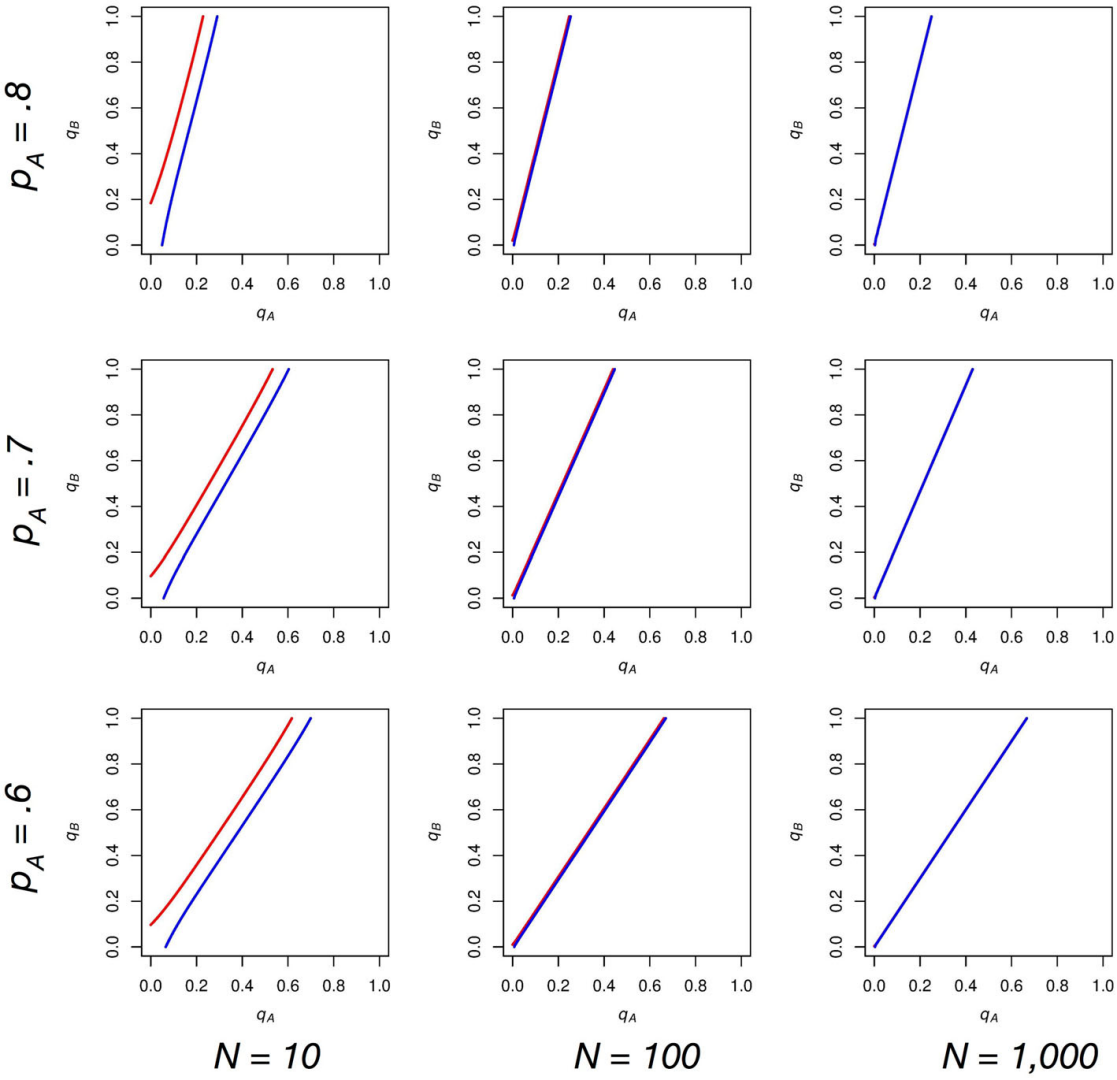
The effect of varying *N* and $p_\mathrm{A}$ on the indifference functions of *A* (red) and *B* (blue) supporters while $c = 0$. These functions never intersect, but approach collinearity as $N \rightarrow \infty$. This is because the probability of a tie approaches 0 as *N* gets large, and with *c* equal to zero, individuals are indifferent between voting and abstaining when candidate *A* and candidate *B* have the same number of expected voters (Color figure online)

In each plot, we show the curve along which *A* supporters are indifferent between voting and abstaining (red) and the curve along which *B* supporters are indifferent between voting and abstaining (blue). The x-axis of each graph is $q_\mathrm{A}$ and the y-axis is $q_\mathrm{B}$, with both of these variables taking values in the range [0,1]. Figure 1 shows the effect of varying *N* and $p_\mathrm{A}$ in our model when we set *c* equal to zero.

As we let *N* grow, *A* and *B*’s indifference curves converge to a single line with slope $p_\mathrm{A}/p_\mathrm{B}$. Let us consider why this is the case. As *N* gets large, the probability of a tie goes to zero, all else equal. Furthermore, with *c* (and thus the impact of the underdog effect) equal to zero, this game simplifies to a game akin to the following: Abstain and get a payoff of zero, vote and get a payoff of 1 if your candidate of choice wins and -1 if your candidate of choice loses. So, supporters of a given party are indifferent between voting and abstaining (their expected payoff for voting is equal to zero) when their likelihoods of winning and losing are approximately equal, or when the expected number of *A* and *B* voters are close to the same. As the number of *A* and *B* voters are binomial variables, the expected number of *A* voters is ${N{ p}}_\mathrm{A}q_\mathrm{A}$, and the expected number of *B* voters is ${N}p_\mathrm{B}q_\mathrm{B}$. We find that these are equal when $q_\mathrm{A} = p_\mathrm{B}$ and $q_B = p_A$, making the expected number of voters for both parties equal to ${N}p_\mathrm{A}p_\mathrm{B}$. With $q_\mathrm{A}$ and $q_\mathrm{B}$ set to these levels, the slope ($\Delta q_\mathrm{B}/\Delta q_\mathrm{A}$) approaches $p_\mathrm{A}/p_\mathrm{B}$. With the probability of a tie approaching zero as we let *N* get large, the indifference curves of *A* and *B* supporters approach the same line with this slope.

Now we want to analyze what turnout states are rationalizable for a given set of parameters. In order to do so, we search not just for Nash equilibria, but for *evolutionarily stable strategies* (*ESS*es). Similar to the work of Sieg and Schulz [39], we consider the learning individual in a voting context, wherein actions that induced pleasure are repeated, and actions that induced punishment are avoided. To show how we determine *ESS*es, we consider the simple case when $N = 10$, $p_\mathrm{A} = .6$, and $c=0$ (bottom left panel of Fig. 1).

**Fig. 2:**
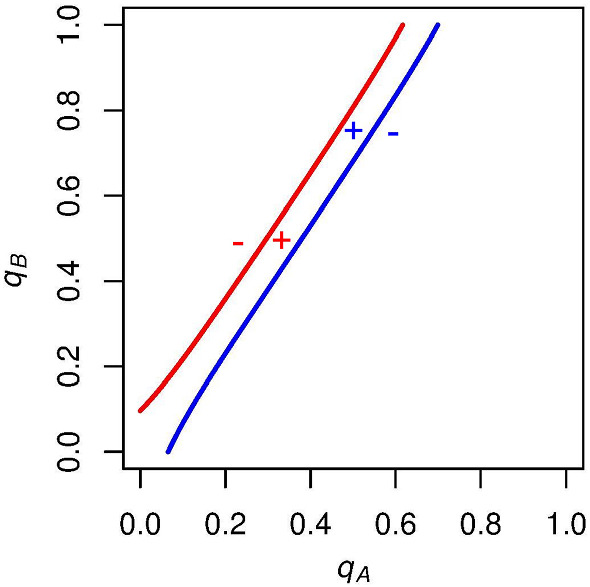
Example graph with expected voting payoff signs. To the right of the red curve, the expected voting payoff for an *A* supporter is positive, and to the left of this curve it is negative. Above the blue curve, the expected voting payoff for a *B* supporter is positive, and below this curve it is negative. Parameters are set to $N = 10$, $p_\mathrm{A} = .6$, and $c = 0$

We first want to consider which regions of the graph correspond to positive or negative expected voting payoffs for both *A* and *B* supporters. Focusing on *A* supporters (red curve), we know that their expected payoff for voting is exactly zero along this curve. If $q_A$ is unilaterally increased from a point on this curve, then candidate *A* is more likely to win the election and so *A* supporters are more likely to get the winning (positive when $c=0$) payoff for voting. Thus, to the right of the red curve, *A* supporters have a positive expected payoff for voting (seen by the red plus in Fig. 2). Analogously, when we decrease $q_A$ from a point on the red curve, we find that candidate *A* is more likely to lose the election, and so *A* supporters are more likely to get the negative payoff for voting when candidate *A* loses. To the left of the red curve then, *A* supporters have a negative expected payoff for voting (seen by the red minus in Fig. 2).

Similarly, we find that when *B* supporters increase $q_B$ from a point on the blue curve, it will increase candidate *B*’s likelihood of winning, and *B* supporters will have a positive expected payoff for voting. Below the blue curve, then, *B* supporters will have a negative expected payoff for voting. These signs can also be seen in Fig. 2

In regions where the expected payoff for voting is positive, groups will want to increase the frequency with which they vote, and in negative regions, they will want to increase the frequency with which they abstain.

As noted earlier, voters are not rational utility maximizers in this context; that is, they do not calculate which decisions yield the best outcomes. Rather, they start with a random choice, and then repeat actions that induced pleasure, and avoid actions that induced penalty. These random initial choices for $q_\mathrm{A}$ and $q_\mathrm{B}$ can lie in 3 different regions that have different implications for voter behavior.

**Fig. 3:**
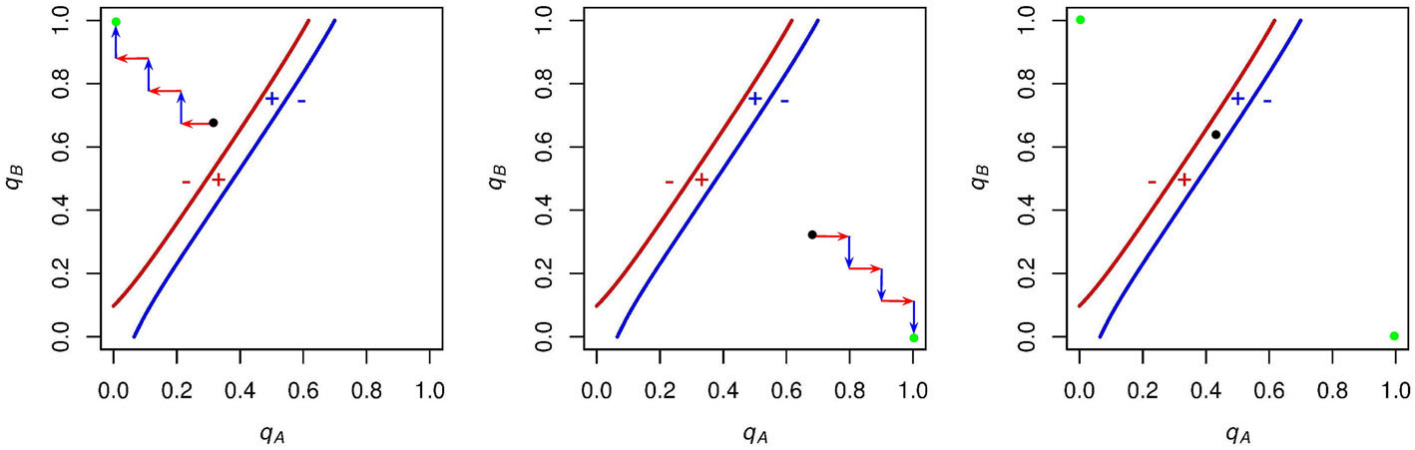
$\mathrm{ESS}$es when $N = 10$, $p_\mathrm{A} = .6$, and $c = 0$. Left panel: If *A* supporters and *B* supporters choose voting probabilities such that the initial state (represented by a black point) is in the top left region of the graph, then turnout will run away to the $\mathrm{ESS}$ (denoted by the green point) of $q_\mathrm{A}=0$, $q_\mathrm{B}=1$. Middle panel: If the initial state is in the bottom right region of the graph, the $\mathrm{ESS}$ will be $q_\mathrm{A}=1, q_\mathrm{B}=0$. Right panel: If the initial state is between these curves, both $q_\mathrm{A}$ and $q_\mathrm{B}$ will increase until the state breaks into one of the first two regions. This will lead to one of the two $\mathrm{ESS}$es from before. Altogether, we have that turnout runs away to either full *A* turnout and no *B* turnout or no *A* turnout and full *B* turnout for this set of parameters, depending on the initial state

If *A* and *B* supporters choose initial $q_\mathrm{A}$ and $q_\mathrm{B}$ values such that the initial state is above *A* supporters’ indifference curves, then in this region *A* supporters get a negative expected payoff for voting, and *B* supporters get a positive expected payoff for voting (Fig. 3, left panel). Consequently, *A* supporters will decrease the likelihood with which they vote (leftward-pointing arrows in the left panel of Fig. 3) and *B* supporters will increase the likelihood with which they vote (upward-pointing arrows in the left panel Fig. 3). This process will iterate, with $q_A$ decreasing and $q_B$ increasing until turnout “runs away” to the stable state of no *A* turnout and full *B* turnout.

If, instead, the initial state is below *B* supporters’ indifference curve, then voting has a positive expected payoff for *A* supporters and a negative expected payoff for *B* supporters (Fig. 3, middle panel). Thus, $q_\mathrm{A}$ will increase and $q_\mathrm{B}$ will decrease until the other stable state of full *A* turnout and no *B* turnout is reached.

Finally, if the initial state is between these curves, then all individuals have a positive expected payoff for voting (Fig. 3, right panel). Both *A* supporters and *B* supporters will increase their voting probabilities until turnout breaks into one of the two original regions, and runs away to the respective stable state of either full *A* turnout and no *B* turnout or no *A* turnout and full *B* turnout. So whenever $c = 0$ and the indifference curves are configured in this way, the two stable states are full *A* turnout and no *B* turnout, or no *A* turnout and full *B* turnout.

**Fig. 4:**
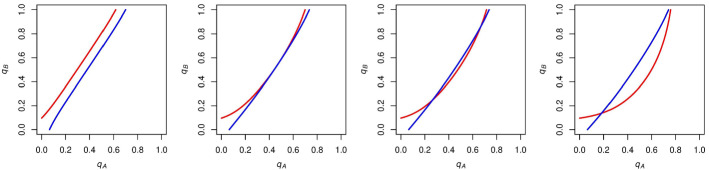
The effect of increasing *c* from zero (left-most panel) but below $1/(p_\mathrm{A} - .5)$ on the configuration of *A* and *B*’s indifference curves (increasing *c* from left to right). At first *A*’s indifference curve is tangent to *B*’s. As we increase *c* further, the curves intersect twice, and as we increase *c* beyond this point, there is just one intersection. These new configurations could give rise to new equilibria and are analyzed below

We now go on to analyze how these indifference curves and their associated stable states change as we alter *c*. Figure 4 shows the effect of increasing *c* from 0 but below $1/(p_\mathrm{A} - .5)$, the value for which the voting payoff for an *A* supporter when candidate *A* wins would become negative. We hold *N* and $p_\mathrm{A}$ constant at 10 and .6 respectively throughout this analysis in order to make our $\mathrm{ESS}$es more visually compelling, but the types of $\mathrm{ESS}$es are the same if we vary either *N* or $p_\mathrm{A}$ within the ranges of *c* that we enumerate later. The effect of changing these parameters while holding *c* constant is analyzed in Sect. 3.2.

**Fig. 5:**
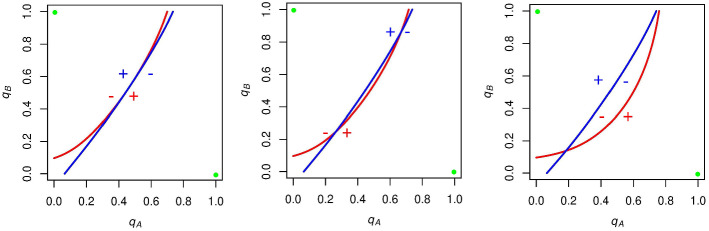
Regardless of the number of intersections between *A* and *B*’s indifference curves, when $0 \le c \le 1/(p_\mathrm{A} - .5)$, the only $\mathrm{ESS}$es (represented by green points) that survive are $q_\mathrm{A} = 1$ (full *A* turnout) and $q_\mathrm{B} = 0$ (no *B* turnout) or $q_\mathrm{A} = 0$ (no *A* turnout) and $q_\mathrm{B} = 1$ (full *B* turnout). Varying *c* within the range $0 \le c \le 1/(p_\mathrm{A} - .5)$ does not affect the stable states that arise (Color figure online)

We now go on to analyze what $\mathrm{ESS}$es are rationalizable in the cases shown in Fig. 4, when there exist intersections (and associated mixed-strategy Nash equilibria) between *A* and *B*’s indifference curves. Figure 5 shows the results of this analysis.

We find that, although these curves have mixed-strategy equilibria, they are unstable: any infinitesimal deviation by either *A* or *B* supporters from these Nash probabilities would give rise to further deviation. In each of these cases, we find that turnout once again runs away to the two stable states from before: either full *A* turnout and no *B* turnout, or no *A* turnout and full *B* turnout.

**Fig. 6:**
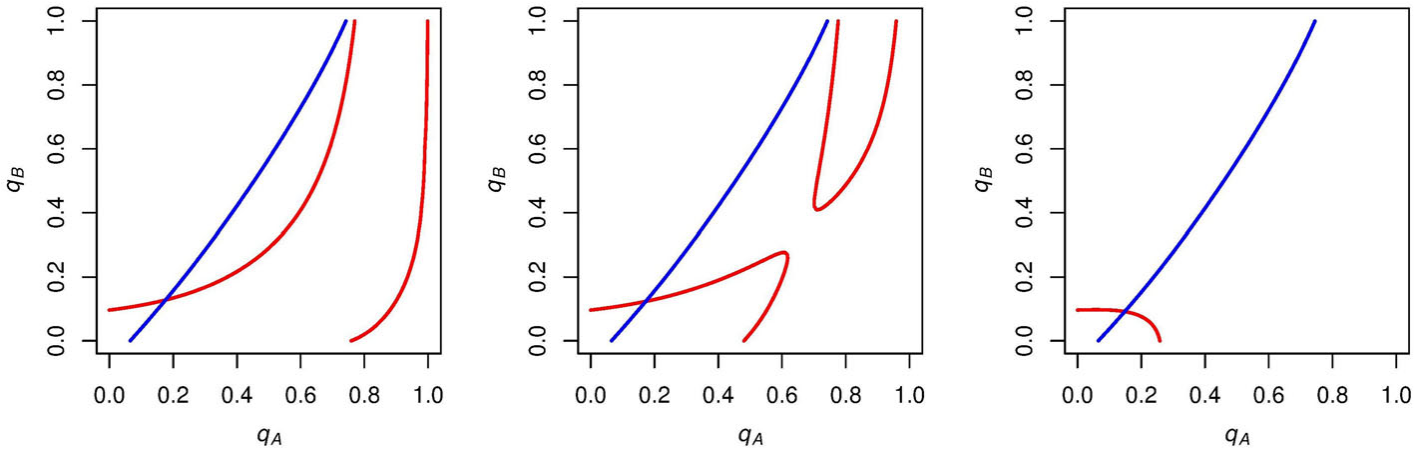
The effect of increasing *c* on the configuration of *A* and *B*’s indifference curves when $1/(p_\mathrm{A} - .5) < c \le 2/(p_\mathrm{A} - .5)$, with *c* is increasing in panels from left to right. The presence of a second curve for *A* supporters as well as the orientation of the *A* curve in the rightmost panel could lead to new equilibria which are analyzed below

We now go on to analyze the effect that increasing *c* beyond $1/(p_\mathrm{A} - .5)$ will have on the configurations and respective turnouts of *A* and *B*’s indifference curves. It is notable that once *c* exceeds $1/(p_\mathrm{A} - .5)$, the payoff for an *A* supporter voting when candidate *A* wins becomes negative. In this situation, the only way that an *A* supporter can get a positive payoff for voting is if the candidates tie, and this individual’s vote is pivotal in preventing a loss for candidate *A*. We first focus on cases when $1/(p_\mathrm{A} - .5) < c \le 2/(p_\mathrm{A} - .5)$, as once *c* exceeds $2/(p_\mathrm{A} - .5)$, the payoff for an *A* supporter voting when candidate *A* wins becomes lower than -1, which is the payoff for an *A* supporter voting when candidate *A* loses. The effect of increasing *c* between $1/(p_\mathrm{A} - .5)$ and $2/(p_\mathrm{A} - .5)$ is shown in Fig. 6.

**Fig. 7:**
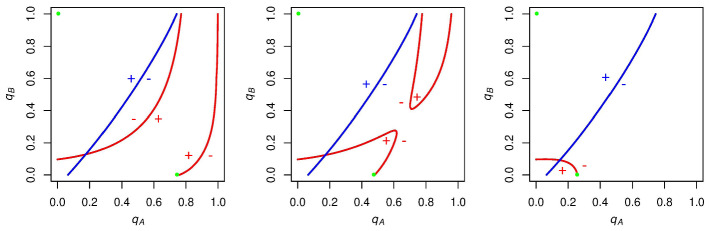
$\mathrm{ESS}$es as we increase *c* when $1/(p_\mathrm{A} - .5) < c \le 2/(p_\mathrm{A} - .5)$. Stable states either have no *A* turnout and full *B* turnout or $\mathrm{some}$ *A* turnout in expectation and no *B* turnout. Expected *A* turnout is decreasing in *c* for the latter stable state

We go on to analyze $\mathrm{ESS}$es for these examples in Fig. 7. It is noteworthy that *A*’s expected voting payoff is not always increasing in $q_\mathrm{A}$ when *c* is in this range. If $q_\mathrm{A}$ is sufficiently high (and $q_\mathrm{B}$ is sufficiently low), then *A* supporters will be likely to win and accordingly get the negative payoff associated with voting and winning. This will compel *A* supporters to abstain more and increase the likelihood of a tied election.

In each of these examples, it is clear that the stable state of no *A* turnout and full *B* turnout still exists, but now the stable state of full *A* turnout and no *B* turnout disappears. Instead, each of these cases has a stable state with *some* *A* turnout in expectation and no *B* turnout. This expected *A* turnout is decreasing in *c* (left to right in Fig. 7).

We find that *A* supporters’ ability to vote with these probabilities strictly between zero and one in equilibrium is supported by the fact that *A* supporters always have a curve that is “stable” when *c* is in this range; that is, if *A* supporters increase $q_\mathrm{A}$ beyond (to the right of) this stable curve, they will get a negative expected payoff from voting and will abstain more by decreasing $q_\mathrm{A}$, moving back toward the curve. If $q_\mathrm{A}$ is decreased beyond (to the left of) this stable curve, *A* supporters will get a positive expected payoff from voting, and will vote more by increasing $q_\mathrm{A}$ back toward this curve.

**Fig. 8:**
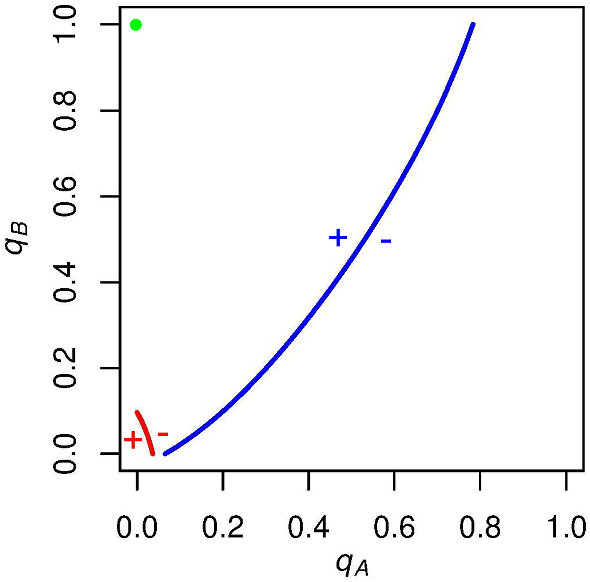
Example $\mathrm{ESS}$ when $c > 2/(p_\mathrm{A} - .5)$. The only stable state has no *A* turnout and full *B* turnout. Thus, when *c* is sufficiently high, *A* turnout is driven to zero by the low payoff for voting received by *A* supporters when candidate *A* wins

In Fig. 8, we analyze what happens when *c* exceeds $2/(p_\mathrm{A} - .5)$. Remember that when *c* takes on such values, *A* supporters’ voting payoff when candidate *A* wins is now less than *A* supporters’ voting payoff when candidate *A* loses (-1). We find that whenever *c* exceeds this value, *A* and *B*’s indifference curves no longer intersect, and the only stable state that remains has no *A* turnout and full *B* turnout.

To summarize, when $0 \le c \le 1/(p_\mathrm{A} - .5)$, there are two stable states: no *A* turnout and full *B* turnout, or full *A* turnout and no *B* turnout. When $1/(p_\mathrm{A} - .5) < c \le 2/(p_\mathrm{A} - .5)$, there are also two stable states: no *A* turnout and full *B* turnout, or *some* *A* turnout (in expectation) and no *B* turnout. Lastly, when $c > 2/(p_A - .5)$, the only stable state that remains has no *A* turnout and full *B* turnout. We now go on to consider what negative levels of *c* might mean qualitatively, and what implications relaxing this assumption might have for voter behavior.

### Turnout When $c < 0$

It is interesting to consider whether having a negative value for *c* makes sense in respect to human behavior. This would mean that those who perceive themselves likely to win (in our case, those in the majority) get an additional benefit for voting when their candidate wins, and those who perceive themselves unlikely to win (in our case, those in the minority) get a penalty on their voting payoff when their candidate wins.

Rather than having an “underdog effect,” wherein people in the minority enjoy their victory more than those in the majority, we now have a “will-of-the-people effect.” In this type of world, voters in the majority get a benefit when their candidate wins for supporting the election of a candidate that more people prefer. Voters in the minority get a penalty when their candidate wins as they may feel some guilt for electing a candidate that is not as well-supported (leaving this voter feeling somewhat selfish).

**Fig. 9:**
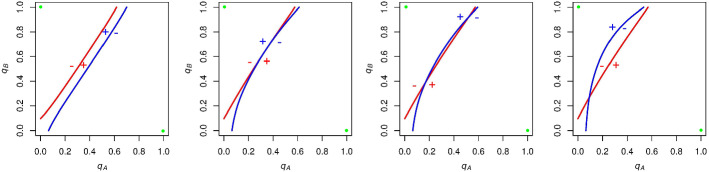
The effect of decreasing *c* (left to right) from zero but not beyond $1/(.5-p_\mathrm{A})$. Regardless of the number of intersections between these indifference curves, there are only two stable states: full *A* turnout and no *B* turnout or no *A* turnout and full *B* turnout. These stable states are the same as those found in Fig. 5

With this framework in mind, we go on to analyze what stable states arise from different negative values of *c*, finding relatively symmetric results to the positive values of *c*. Figure 9 shows the results of decreasing *c* from zero but not beyond $1/(.5-p_\mathrm{A})$, the point at which *B* supporters’ voting payoff when candidate *B* wins becomes negative. As we decrease *c* (left to right in Fig. 9), we find that, although we get a varying number of Nash equilibria, we end up with the two stable states of full *A* turnout and no *B* turnout or no *A* turnout and full *B* turnout that we found when $0 \le c < 1/(p_\mathrm{A}-.5)$.

Similar to the positive *c* case, we find that our $\mathrm{ESS}$es change once *c* exceeds $1/(.5-p_\mathrm{A})$. Beyond this point, the voting payoff for a *B* supporter when candidate *B* wins becomes negative, meaning that *B* supporters can only get a positive payoff from voting if the result of the election is a tie.

This leads to a symmetric finding to the positive *c* cases: when $2/(.5 - p_\mathrm{A}) \le c < 1/(.5 - p_\mathrm{A})$, *B* supporters have an indifference curve that is stable. If *B* supporters deviate from this curve by increasing $q_\mathrm{B}$, they will receive a negative payoff for voting, and will abstain more, decreasing $q_\mathrm{B}$ toward this curve. If they deviate from this curve by decreasing $q_\mathrm{B}$, they will receive a positive payoff from voting, will vote more, and increase $q_\mathrm{B}$ back toward this curve.

**Fig. 10:**
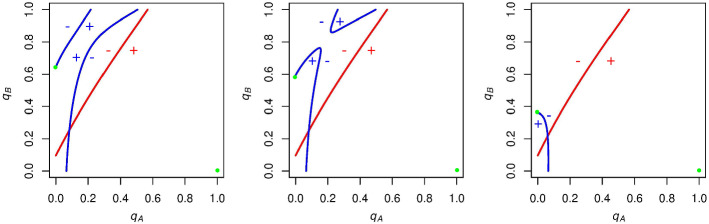
The effect of decreasing *c* (left to right) when $2/(.5 - p_\mathrm{A}) \le c < 1/(.5 - p_\mathrm{A})$. In each of these cases, there are two stable states: full *A* turnout and no *B* turnout, or no *A* turnout and some *B* turnout in expectation. Expected *B* turnout for the latter case is decreasing in the magnitude of *c*. This result is analogous to the result in Fig. 7

This allows for equilibria in which supporters from one bloc vote with a probability strictly between zero and one. Once again, we find that this voting probability is decreasing in the magnitude of *c* (in the negative direction, in this case). The results of this analysis can be seen in Fig. 10.

**Fig. 11:**
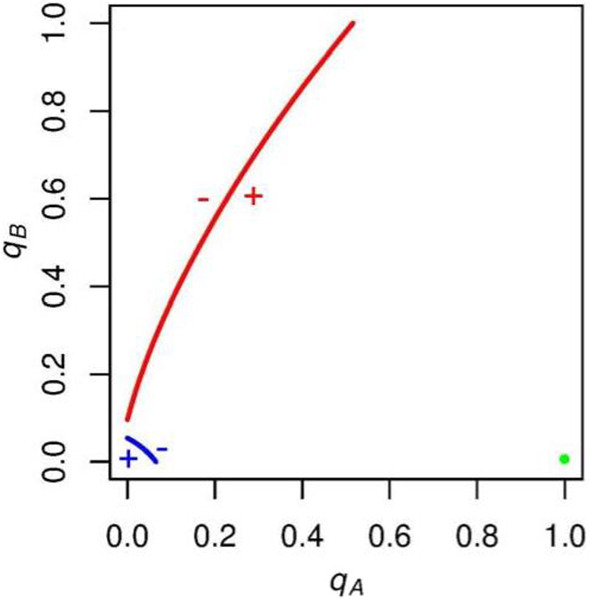
An example graph for when $c < 2/(.5 - p_\mathrm{A})$. When *c* is less than this value, the indifference curves no longer intersect, and the only stable state has full *A* turnout and no *B* turnout. Thus, when c is sufficiently low, *B* turnout is driven to zero by the low payoff for voting received by *B* supporters when candidate *B* wins. This is analogous to the result in Fig. 8

When we decrease *c* beyond $2/(.5 - p_\mathrm{A})$, *B* supporters’ payoff for voting when candidate *B* wins becomes less than the payoff for voting when candidate *B* loses (-1). In this case, as with its positive analog, *A* and *B*’s indifference curves no longer intersect, and the stable state with partial turnout in expectation disappears (Fig. 11).

At this point we have covered all of the different ranges for *c* and analyzed what $\mathrm{ESS}$es exist in each case. A summary of this analysis can be found in Table 1.

**Table 1:** $\mathrm{ESS}$es Summary

*c* Range	$\mathrm{ESS}$ 1	$\mathrm{ESS}$ 2
$c > 2/(p_\mathrm{A}-.5)$	$q_A = 0, q_\mathrm{B} = 1$	
$1/(p_\mathrm{A}-.5) < c \le 2/(p_\mathrm{A}-.5)$	$q_\mathrm{A} = 0, q_\mathrm{B} = 1$	$0<q_\mathrm{A}<1, q_\mathrm{B} = 0$
$1/(.5-p_\mathrm{A}) \le c \le 1/(p_\mathrm{A}-.5)$	$q_\mathrm{A} = 0, q_\mathrm{B} = 1$	$q_\mathrm{A} = 1, q_\mathrm{B} = 0$
$2/(.5-p_\mathrm{A}) \le c < 1/(.5-p_\mathrm{A})$	$q_\mathrm{A} = 1, q_\mathrm{B} = 0$	$q_\mathrm{A}=0, 0<q_\mathrm{B}<1$
$c < 2/(.5-p_\mathrm{A})$	$q_\mathrm{A} = 1, q_\mathrm{B} = 0$	

### Comparative Statics

Another thing worth considering is the effect that changing either the size of the population (*N*) or the partisan spread ($p_\mathrm{A}$ vs. $p_\mathrm{B}$) would have on voter behavior. Remember that we have thus far focused on the simplistic case of a 6 vs. 4 person election for ease of analysis. While we have analyzed the effect of varying *c* on turnout for this particular case, we want to see how varying these other parameters might affect voter behavior.

Table 1 shows that many of our stable states are of the “all or nothing” variety, with either full turnout or no turnout from the supporters of each party. We find that these stable states remain regardless of changes to *N* and $p_\mathrm{A}$; whenever *c* is within the intervals outlined in Table 1, the “all or nothing” stable states are not altered by changes in either the number of individuals in the electorate or the partisan spread of these individuals.

**Fig. 12:**
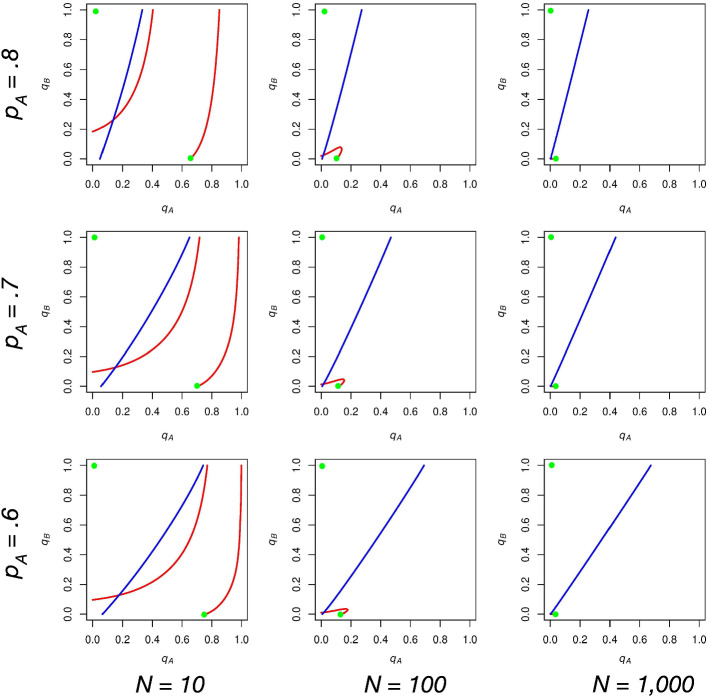
The effect of varying *N* and $p_\mathrm{A}$ while holding *c* constant at $10,001/(10,000(p_\mathrm{A}-.5))$. The $\mathrm{ESS}$ when *A* supporters vote with a probability strictly between zero and one has expected turnout that is decreasing in *N* as well as $p_\mathrm{A}$, ceteris paribus

With this in mind, we turn to the cases when one of the parties votes with a probability strictly between zero and one. We focus on the case when $1/(p_\mathrm{A}-.5) < c \le 2/(p_\mathrm{A}-.5)$ and there exists an $\mathrm{ESS}$ in which $0<q_\mathrm{A}<1$, but we will discuss the comparative statics for the negative analog of this case as well. Figure 12 provides a visual representation of how this $\mathrm{ESS}$ changes as we vary *N* and $p_\mathrm{A}$.

We find that both *N* and $p_\mathrm{A}$ affect expected turnout for these equilibria. The more striking influence is this “size effect” predicted by our model, wherein expected turnout is decreasing (quite rapidly) in *N*.

This makes sense when we consider the range of *c* that we are considering. When $1/(p_\mathrm{A}-.5) < c \le 2/(p_\mathrm{A}-.5)$, the only way *A* supporters can get a positive payoff for voting is if the result of the election is a tie. In order to drive turnout away from zero in expectation for *A* supporters, then, a tie must be sufficiently likely to occur; as we increase *N* while holding *c* and $p_\mathrm{A}$ constant, the probability of the election resulting in a tie approaches zero, and thus *A* supporters become unable to vote at high rates. Focusing on the bottom row of Fig. 12, one can imagine how the likelihood of a tie may change from a 6 versus 4 person election to a 60 versus 40 person election to a 600 versus 400 person election.

Furthermore, we find this size effect to hold true when looking at the negative analog of this case. When $2/(.5-p_\mathrm{A}) \le c < 1/(.5-p_\mathrm{A})$ and we focus on the $\mathrm{ESS}$ in which $0<q_\mathrm{B}<1$, expected turnout is also decreasing in *N*.

Back to the positive case, when viewing the impact that varying $p_\mathrm{A}$ has on expected turnout, it is less dramatic than the impact of varying *N*. It is nonetheless the case that expected turnout is decreasing in $p_\mathrm{A}$. Let us consider why this is so.

If we hold *N* and *c* constant while increasing $p_\mathrm{A}$, we are increasing the partisan spread, and making candidate *A* more likely to win, all else equal. As *A* supporters prefer a tie to candidate *A* winning (as their vote is pivotal), at high $p_\mathrm{A}$’s, *A* supporters will have a decrease in the $q_\mathrm{A}$-cutpoint at which their expected payoff for voting switches from positive to negative. This cutpoint (when $q_\mathrm{B}$ is equal to zero) is exactly the $\mathrm{ESS}$ we are discussing. Therefore, we find expected turnout to be decreasing in $p_\mathrm{A}$ for this type of equilibrium.

Once again, we consider whether this comparative static holds for the negative analog of this case. In the negative *c* scenario, it is *B* supporters who will only get a positive payoff from voting if there is a tie. If we consider the $q_\mathrm{B}$-cutpoint at which *B* supporters’ expected payoff switches from positive (tie is sufficiently likely) to negative (candidate *B* winning is sufficiently likely), we should find this point increasing in $p_\mathrm{A}$. When *B* supporters comprise only a very small proportion of the electorate (large $p_\mathrm{A}$), then even at high $q_\mathrm{B}$ levels, it is less likely that candidate *B* will win in comparison to when $p_\mathrm{A}$ is smaller. Thus, we find the opposite $p_\mathrm{A}$ effect in the negative case: expected turnout is increasing in $p_\mathrm{A}$.

While we do not find a consistent effect of partisan split on expected voter turnout, we do find that, whenever *N* affects our equilibrium state, expected voter turnout monotonically decreases in *N*. We go on to see if this is consistent with real voter behavior.

## Data

In general, this size effect, in which turnout and electorate size are negatively correlated, has been found to be consistent with voter behavior in a myriad of contexts. While many cite this as evidence that Downs’ formulation of the instrumental voter is correct, others have interpreted the somewhat weak but consistent correlation between electorate size and turnout as evidence that the instrumentality of one’s vote is only *part* of the motivation for voting, an interpretation in agreement with our model.

For example, Levine and Palfrey [23] conduct an experiment in order to examine how voter turnout is affected by different variables, electorate size among them. Using ‘electorate sizes’ no larger than 51, they find strong evidence that size and turnout are negatively correlated. They still find, though, that the size effect cannot entirely explain voting if we consider the solely instrumental voter; rather, they propose that a blend of rationality models may be necessary to fully explain voter behavior.

**Fig. 13:**
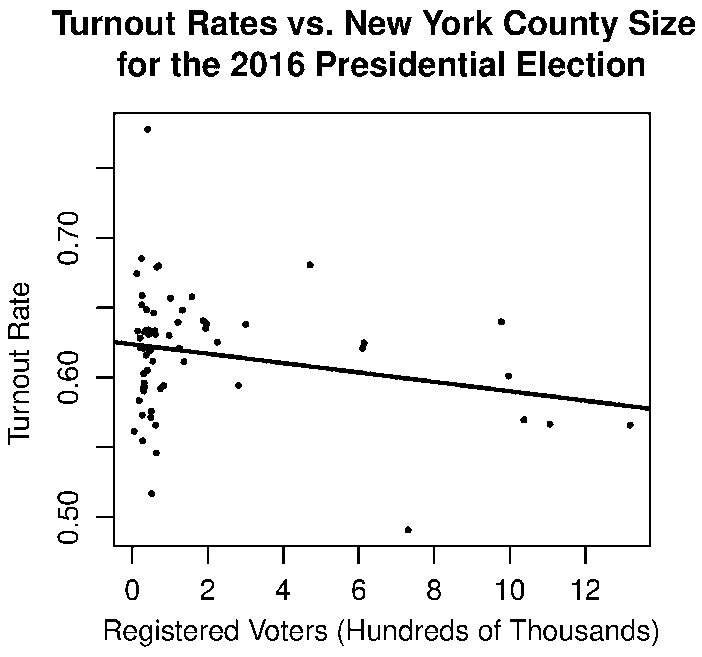
We find that turnout is decreasing in *N*, similar to the comparative statics predicted by our model. We use the number of registered voters as reported by the Board of Elections [4] and the vote count as reported by OpenDataSoft [29]. Turnout is calculated as the number of votes in the county divided by the number of registered voters in the county as of April 1, 2016. Even when the probability of a tie is quite small, this size effect seems to persist in voter behavior

Observational studies support this size effect as well. Breux, Couture, and Goodman [8] find that electorate size is the most important factor in motivating turnout in the municipal elections they study, but that this impact is more prevalent in relatively small electorates.

Still, even in elections with large electorates (gubernatorial elections in this case), the negative correlation between electorate size and turnout persists, although it is relatively weak [2]. This is an interesting finding, as our model predicts that this size effect becomes relatively negligible (as expected turnout approaches zero quickly) as *N* gets large. As the probability of a tie becomes staggeringly small in large electorates, we would expect turnout to decrease almost imperceptibly in *N* for large electorates.

We analyze a dataset with smaller *N*’s than gubernatorial races- the New York State turnout for the 2016 presidential election by county (Fig. 13). Plotting a least squares regression line over these points, we find that the relationship between voter turnout and electorate size has the same sign that our model predicts. This relationship is somewhat weak, again suggesting that individuals do not only consider whether or not their vote will be pivotal when they make the decision of whether or not to vote.

Still, it is notable that this relationship between electorate size and turnout persists even when we are dealing with electorates that our model predicts would have expected turnout rates extremely close to zero. We go on to propose some possible extensions to our base model that reconciles this, as well as another common turnout trend, with our model.

## Extensions

Although our model was successful in the direction of its comparative static predictions, it is certainly not without its shortcomings. Our model still underestimates turnout for large electorates, and does not predict any stable states with expected turnout from both parties. While we believe that the formulation of voter motives posited by our base model is powerful, allowing for more variation in our parameters leads to different rationalizable equilibria that we explore below, some of which we find to be more descriptively accurate than our results above.

First, we observe the effect of relaxing the assumption that the partisan split of the electorate is common knowledge, as this is more often than not the case in the real world. A variety of factors can lead to misperceptions regarding the partisan split of an electorate. Some people are misguided about this from a lack of information, some from conflicting information, and some from the spread of false information, an issue ever so present in the most recent election cycles.

If we let $\tilde{p_\mathrm{A}}$ equal the perceived proportion of the population that supports candidate *A*, with $\tilde{p_\mathrm{B}}$ defined analogously, we can analyze how these perceptions can impact voter turnout. Consistent with our finding from earlier, in the $\mathrm{ESS}$ in which $0<q_\mathrm{A}<1$, we find that turnout is decreasing in $p_\mathrm{A}$. Thus, allowing *c* to be a consistent function of $\tilde{p_\mathrm{A}}$, we find that turnout is higher in expectation whenever $\tilde{p_\mathrm{A}} < p_\mathrm{A}$. We also found that, for the $\mathrm{ESS}$ in which $0<q_\mathrm{B}<1$, turnout is increasing in $p_\mathrm{A}$, and so is decreasing in $p_\mathrm{B}$. In this case, expected turnout is higher whenever $\tilde{p_\mathrm{B}} < p_\mathrm{B}$.

This finding is both descriptive and prescriptive. As we’ve noted, people often vote at higher rates than our model predicts; however, we find that allowing misperceptions about the partisan split of a population might provide an explanation for behavior that is seemingly irrational. If individuals in the majority feel that the partisan split is closer than in reality it is, this will compel them to turn out at higher rates than they would if they knew the true distribution of preferences in the electorate. If individuals in the minority think that a win is not likely, this will motivate more of these individuals to vote as they know every vote for their candidate is more vital to preventing a loss.

As a disclaimer, we do not advocate the spread of false information by politicians or anyone for that matter. However, we do find that bending the perceptions of a support base may have tangible impacts on voter turnout. If a candidate has a majority of the support in an election (particularly a large majority), then she runs the risk of her supporters free-riding and abstaining while a fraction of supporters vote and prop up the possibility of a tied election. Thus, we find that it may be beneficial for this candidate to downplay the majority support she holds when talking to her electorate. By convincing supporters that the race is closer than it actually is, she can avoid this free-rider problem and be more likely to secure her (arguably well-deserved) victory.

**Fig. 14:**
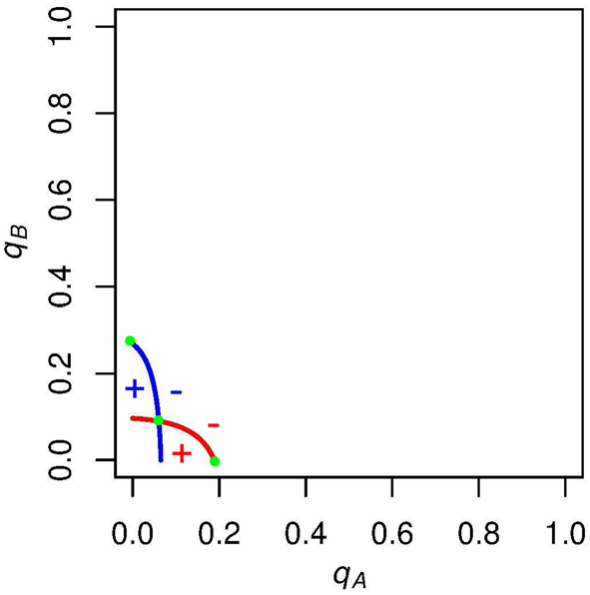
$\mathrm{ESS}$es when we allow *c* diversity in our model. In this simple case ($N = 10$, $p_\mathrm{A} = .6$, $c_\mathrm{A} = 12$, $c_\mathrm{B} = -12$), we find three $\mathrm{ESS}$es. Most notably there exists an $\mathrm{ESS}$ with positive expected turnout from both parties. While turnout is low, it is encouraging that our model accords with real voting behavior in this scenario, predicting a stable state of non-zero turnout in expectation from both parties

On the other side of this, there lies a strategy that a candidate with minority support might find beneficial for spurring turnout from his support base. When a candidate has minority support, it will behoove him to downplay the size of this support. This may seem counterintuitive, as one would think that emphasizing how close the race is would motivate turnout. However, our model finds that whenever a candidate overestimates the magnitude of one’s support to his base (implementing a $\tilde{p_\mathrm{B}}$ that is larger than $p_\mathrm{B}$), he runs the risk of his supporters abstaining more. A candidate in this scenario would find it in his best interest to downplay the size of his support, compelling supporters to view their vote as more integral to preventing the loss of their candidate of choice.

While we offer the misperception of $p_\mathrm{A}$ and $p_\mathrm{B}$ as a possible solution to the over-voting we see in electoral behavior, our model is still in discord with the fact that, in electoral politics, turnout rates are almost always strictly between zero and one for supporters of both candidates. However, we find that relaxing the assumption that the electorate for a given election has a universal *c* reconciles our model with this phenomenon. Allowing for a (potentially) different $c_j$ for supporters of candidate $j \in {(A, B)}$ leads to types of $\mathrm{ESS}$es that were not seen with the base model.

For example, consider a case when $c_\mathrm{A}$ is positive and $c_\mathrm{B}$ is negative. Qualitatively, this means that *A* voters receive a penalty for being in the majority when candidate *A* wins (as this outcome seems inevitable anyways), and *B* voters receive a penalty for being in the minority when candidate *B* wins (as they help elect a candidate that is not well-supported by the electorate). Both blocs have voting payoffs when their candidate of choice wins that are decreasing in $p_\mathrm{A}$.

When we set $c_\mathrm{A}$ and $c_\mathrm{B}$ to levels such that the payoff for voting when one’s candidate of choice wins is less than the payoff for abstaining but greater than the payoff for voting when one’s candidate of choice loses (between –1 and 0), we can find $\mathrm{ESS}$es that could not be obtained using our base model alone. Figure 14 shows one such case.

Based on these new parameters, our model predicts three distinct $\mathrm{ESS}$es: one in which $0<q_\mathrm{A}<1$ and $q_\mathrm{B}=0$, one in which $q_\mathrm{A}=0$ and $0<q_\mathrm{B}<1$, and one in which $0<q_\mathrm{A}<1$ and $0<q_\mathrm{B}<1$. This third $\mathrm{ESS}$ is notable because it reconciles our model with real electoral behavior. In nearly all elections, supporters of each party vote with turnout rates strictly between zero and one; while our base model is inconsistent with this finding, introducing *c* diversity between supporters of different candidates into our payoff structure brings our model in accordance with such behavior.

## Conclusion

While many models have attempted to overcome Downs’ original formulation of the voter problem, our model blends instrumental and expressive voting theories, allows for learning in the electorate, and introduces partisan asymmetry in a way that other models do not. We introduce a new variable, *c*, to account for the extent to which a given population does or does not consider the “underdog effect” when individuals make the decision to vote for their candidate of choice or abstain.

We find that changes in this variable compel different types of $\mathrm{ESS}$es. Depending on the range of *c*, our model predicts either one party turning out to vote in full and the other party abstaining in full, or one party partially turning out to vote in expectation and the other party abstaining in full. In the latter case, expected turnout is decreasing in electorate size as well as the relative magnitude of the party that is expected to vote.

This size effect is well-documented in turnout literature, with turnout decreasing in electorate size even when the probability that one’s vote is pivotal is extremely small. An analysis of turnout in New York State counties from the 2016 presidential election agrees with this finding.

While our model predicts turnout that is smaller than we often see in large electorates, relaxing some of our preliminary assumptions may bring our model more in accordance with true voter behavior. Specifically, we find that when individuals have misperceptions about the partisan split of an electorate (and, by proxy, the likelihood of affecting the outcome), turnout can be higher than our base model predicts. Furthermore, we find that, while our base model does not predict positive turnout from both support blocs in equilibrium (a phenomenon common in electoral politics), introducing *c* diversity into our model allows this to exist. We find the incorporation of both partisan misperception and *c* diversity into our model to be especially promising in explaining and predicting voter turnout moving forward.

## Supplementary Information

Below is the link to the electronic supplementary material.

Supplementary material 1 (pdf 325 KB)

Supplementary material 2 (csv 248 KB)

## Data Availability

The datasets analyzed during the current study are available at the following two sources: https://www.elections.ny.gov/NYSBOE/enrollment/county/county_apr16.pdfhttps://public.opendatasoft.com/explore/dataset/usa-2016-presidential-election-by-county/table/?disjunctive.state&refine.state=New+York.

## Declarations

### Code availability

R code used to generate figures is available on GitHub at https://github.com/camguage/Asymmetric-Partisan-Voter-Turnout-Games or available upon request.
