# Supplementary material for: Asymmetric Partisan Voter Turnout Games
Source: Dyn Games Appl. 2021 Apr 16;11(4):738–58. doi: 10.1007/s13235-021-00384-1 (PMC8542561; doi:10.1007/s13235-021-00384-1)
Supplement: Supplementary file 1 — Supplementary material 1 (pdf 325 KB) [file 13235_2021_384_MOESM1_ESM.pdf]

NYSVoter Enrollment by County, Party Affiliation and Status
Voters Registered as of April 1, 2016

REGION	COUNTY	STATUS	DEM	REP	CON	GRE	WOR	IND	WEP	REF	OTH	BLANK	TOTAL
Outside NYC	Albany	Active	88,623	34,944	2,939	487	583	9,271	29	1	195	39,172	176,244
Outside NYC	Albany	Inactive	10,730	2,971	310	90	116	1,119	1	0	107	5,087	20,531
Outside NYC	Albany	Total	99,353	37,915	3,249	577	699	10,390	30	1	302	44,259	196,775
Outside NYC	Allegany	Active	5,869	12,217	442	75	127	1,246	1	1	16	4,509	24,503
Outside NYC	Allegany	Inactive	401	537	20	6	17	102	0	0	3	386	1,472
Outside NYC	Allegany	Total	6,270	12,754	462	81	144	1,348	1	1	19	4,895	25,975
Outside NYC	Broome	Active	40,835	40,667	1,560	334	596	5,949	18	4	87	20,997	111,047
Outside NYC	Broome	Inactive	5,446	3,176	130	80	117	896	1	0	30	4,145	14,021
Outside NYC	Broome	Total	46,281	43,843	1,690	414	713	6,845	19	4	117	25,142	125,068
Outside NYC	Cattaraugus	Active	14,573	17,010	1,109	114	281	2,249	9	0	11	8,884	44,240
Outside NYC	Cattaraugus	Inactive	1,915	1,811	129	22	66	402	0	0	9	1,686	6,040
Outside NYC	Cattaraugus	Total	16,488	18,821	1,238	136	347	2,651	9	0	20	10,570	50,280
Outside NYC	Cayuga	Active	14,745	16,299	1,232	150	235	2,436	2	1	13	9,517	44,630
Outside NYC	Cayuga	Inactive	1,341	1,122	85	14	36	300	0	0	3	1,091	3,992
Outside NYC	Cayuga	Total	16,086	17,421	1,317	164	271	2,736	2	1	16	10,608	48,622
Outside NYC	Chautauqua	Active	25,659	24,934	1,947	161	458	4,716	3	2	67	17,932	75,879
Outside NYC	Chautauqua	Inactive	2,340	1,835	155	30	61	538	0	0	14	2,261	7,234
Outside NYC	Chautauqua	Total	27,999	26,769	2,102	191	519	5,254	3	2	81	20,193	83,113
Outside NYC	Chemung	Active	15,435	20,009	777	104	274	3,092	2	1	21	10,110	49,825
Outside NYC	Chemung	Inactive	1,437	1,397	68	12	34	318	0	0	3	1,156	4,425
Outside NYC	Chemung	Total	16,872	21,406	845	116	308	3,410	2	1	24	11,266	54,250

NYSVoter Enrollment by County, Party Affiliation and Status
Voters Registered as of April 1, 2016

REGION	COUNTY	STATUS	DEM	REP	CON	GRE	WOR	IND	WEP	REF	OTH	BLANK	TOTAL
Outside NYC	Chenango	Active	7,200	12,269	495	133	179	1,645	5	0	16	6,335	28,277
Outside NYC	Chenango	Inactive	659	774	33	14	36	154	0	0	3	727	2,400
Outside NYC	Chenango	Total	7,859	13,043	528	147	215	1,799	5	0	19	7,062	30,677
Outside NYC	Clinton	Active	16,842	14,183	512	90	250	3,225	3	0	0	10,670	45,775
Outside NYC	Clinton	Inactive	1,390	850	27	19	25	355	0	0	0	1,216	3,882
Outside NYC	Clinton	Total	18,232	15,033	539	109	275	3,580	3	0	0	11,886	49,657
Outside NYC	Columbia	Active	13,037	11,774	1,091	181	211	2,711	8	1	22	10,724	39,760
Outside NYC	Columbia	Inactive	1,241	724	83	24	32	323	0	0	5	1,007	3,439
Outside NYC	Columbia	Total	14,278	12,498	1,174	205	243	3,034	8	1	27	11,731	43,199
Outside NYC	Cortland	Active	8,448	9,674	447	95	131	1,535	3	0	5	6,371	26,709
Outside NYC	Cortland	Inactive	1,192	862	41	18	33	238	0	0	1	1,474	3,859
Outside NYC	Cortland	Total	9,640	10,536	488	113	164	1,773	3	0	6	7,845	30,568
Outside NYC	Delaware	Active	7,214	11,543	493	107	112	1,580	2	0	15	5,281	26,347
Outside NYC	Delaware	Inactive	716	715	34	9	16	160	0	0	3	632	2,285
Outside NYC	Delaware	Total	7,930	12,258	527	116	128	1,740	2	0	18	5,913	28,632
Outside NYC	Dutchess	Active	58,020	50,841	3,637	473	765	10,171	28	35	114	47,061	171,145
Outside NYC	Dutchess	Inactive	6,438	3,772	233	63	109	1,071	0	1	21	4,742	16,450
Outside NYC	Dutchess	Total	64,458	54,613	3,870	536	874	11,242	28	36	135	51,803	187,595
Outside NYC	Erie	Active	276,813	146,502	13,398	1,558	2,961	27,821	50	9	460	100,809	570,381
Outside NYC	Erie	Inactive	21,698	8,544	621	177	326	2,214	0	0	57	9,483	43,120
Outside NYC	Erie	Total	298,511	155,046	14,019	1,735	3,287	30,035	50	9	517	110,292	613,501

NYSVoter Enrollment by County, Party Affiliation and Status
Voters Registered as of April 1, 2016

REGION	COUNTY	STATUS	DEM	REP	CON	GRE	WOR	IND	WEP	REF	OTH	BLANK	TOTAL
Outside NYC	Essex	Active	6,338	10,789	213	84	52	1,750	1	0	9	4,395	23,631
Outside NYC	Essex	Inactive	717	944	24	17	20	279	0	0	6	669	2,676
Outside NYC	Essex	Total	7,055	11,733	237	101	72	2,029	1	0	15	5,064	26,307
Outside NYC	Franklin	Active	9,624	8,457	343	59	94	1,603	1	0	3	4,662	24,846
Outside NYC	Franklin	Inactive	810	637	34	15	23	229	0	0	1	715	2,464
Outside NYC	Franklin	Total	10,434	9,094	377	74	117	1,832	1	0	4	5,377	27,310
Outside NYC	Fulton	Active	7,667	15,536	537	67	160	1,657	4	2	6	5,796	31,432
Outside NYC	Fulton	Inactive	424	550	23	10	14	105	0	0	0	423	1,549
Outside NYC	Fulton	Total	8,091	16,086	560	77	174	1,762	4	2	6	6,219	32,981
Outside NYC	Genesee	Active	9,065	15,541	941	93	211	1,879	1	0	64	8,087	35,882
Outside NYC	Genesee	Inactive	498	628	40	9	15	125	0	0	6	517	1,838
Outside NYC	Genesee	Total	9,563	16,169	981	102	226	2,004	1	0	70	8,604	37,720
Outside NYC	Greene	Active	6,927	11,599	822	87	143	1,876	1	1	1	7,394	28,851
Outside NYC	Greene	Inactive	747	859	71	26	17	230	0	0	0	843	2,793
Outside NYC	Greene	Total	7,674	12,458	893	113	160	2,106	1	1	1	8,237	31,644
Outside NYC	Hamilton	Active	877	2,493	70	9	6	234	0	0	3	553	4,245
Outside NYC	Hamilton	Inactive	111	287	9	1	1	45	0	0	2	86	542
Outside NYC	Hamilton	Total	988	2,780	79	10	7	279	0	0	5	639	4,787
Outside NYC	Herkimer	Active	10,007	17,573	659	96	130	2,370	3	0	9	6,311	37,158
Outside NYC	Herkimer	Inactive	841	1,089	48	9	20	237	0	0	1	682	2,927
Outside NYC	Herkimer	Total	10,848	18,662	707	105	150	2,607	3	0	10	6,993	40,085

NYSVoter Enrollment by County, Party Affiliation and Status
Voters Registered as of April 1, 2016

REGION	COUNTY	STATUS	DEM	REP	CON	GRE	WOR	IND	WEP	REF	OTH	BLANK	TOTAL
Outside NYC	Jefferson	Active	15,118	22,605	831	132	206	2,991	3	0	23	11,510	53,419
Outside NYC	Jefferson	Inactive	2,735	2,847	120	26	55	610	0	0	8	2,926	9,327
Outside NYC	Jefferson	Total	17,853	25,452	951	158	261	3,601	3	0	31	14,436	62,746
Outside NYC	Lewis	Active	4,203	8,491	274	32	61	826	0	0	3	2,715	16,605
Outside NYC	Lewis	Inactive	287	451	17	2	3	69	0	0	0	238	1,067
Outside NYC	Lewis	Total	4,490	8,942	291	34	64	895	0	0	3	2,953	17,672
Outside NYC	Livingston	Active	10,010	16,443	886	136	145	1,806	4	1	49	8,128	37,608
Outside NYC	Livingston	Inactive	778	815	52	15	18	177	0	0	7	864	2,726
Outside NYC	Livingston	Total	10,788	17,258	938	151	163	1,983	4	1	56	8,992	40,334
Outside NYC	Madison	Active	11,051	15,633	880	131	233	2,594	3	0	37	9,268	39,830
Outside NYC	Madison	Inactive	1,059	1,032	66	19	20	267	0	0	3	896	3,362
Outside NYC	Madison	Total	12,110	16,665	946	150	253	2,861	3	0	40	10,164	43,192
Outside NYC	Monroe	Active	173,745	125,888	8,096	1,189	1,485	20,157	31	4	481	104,017	435,093
Outside NYC	Monroe	Inactive	16,552	7,271	449	135	200	1,784	1	0	59	8,873	35,324
Outside NYC	Monroe	Total	190,297	133,159	8,545	1,324	1,685	21,941	32	4	540	112,890	470,417
Outside NYC	Montgomery	Active	9,121	9,408	659	60	120	1,530	3	0	21	6,142	27,064
Outside NYC	Montgomery	Inactive	776	632	62	10	24	148	0	0	2	705	2,359
Outside NYC	Montgomery	Total	9,897	10,040	721	70	144	1,678	3	0	23	6,847	29,423
Outside NYC	Nassau	Active	369,720	320,719	10,664	1,535	2,364	35,351	103	14	303	222,479	963,252
Outside NYC	Nassau	Inactive	13,989	10,563	345	76	78	1,366	0	0	7	7,741	34,165
Outside NYC	Nassau	Total	383,709	331,282	11,009	1,611	2,442	36,717	103	14	310	230,220	997,417

NYSVoter Enrollment by County, Party Affiliation and Status
Voters Registered as of April 1, 2016

REGION	COUNTY	STATUS	DEM	REP	CON	GRE	WOR	IND	WEP	REF	OTH	BLANK	TOTAL
Outside NYC	Niagara	Active	51,767	42,326	3,120	427	1,188	6,520	14	29	90	22,874	128,355
Outside NYC	Niagara	Inactive	1,765	1,119	75	21	50	215	0	0	3	1,051	4,299
Outside NYC	Niagara	Total	53,532	43,445	3,195	448	1,238	6,735	14	29	93	23,925	132,654
Outside NYC	Oneida	Active	43,087	47,378	2,037	257	515	7,360	11	2	72	23,593	124,312
Outside NYC	Oneida	Inactive	4,511	3,811	184	39	83	1,020	0	0	6	3,211	12,865
Outside NYC	Oneida	Total	47,598	51,189	2,221	296	598	8,380	11	2	78	26,804	137,177
Outside NYC	Onondaga	Active	99,484	80,943	4,794	943	1,253	14,048	24	12	257	69,380	271,138
Outside NYC	Onondaga	Inactive	12,561	5,907	380	147	222	1,751	1	0	41	8,647	29,657
Outside NYC	Onondaga	Total	112,045	86,850	5,174	1,090	1,475	15,799	25	12	298	78,027	300,795
Outside NYC	Ontario	Active	19,460	26,075	1,376	219	213	3,790	11	1	20	16,068	67,233
Outside NYC	Ontario	Inactive	812	923	57	14	19	221	0	0	2	830	2,878
Outside NYC	Ontario	Total	20,272	26,998	1,433	233	232	4,011	11	1	22	16,898	70,111
Outside NYC	Orange	Active	75,896	68,087	4,142	571	1,061	11,260	23	8	124	47,815	208,987
Outside NYC	Orange	Inactive	6,196	4,229	262	68	115	1,012	0	0	11	4,191	16,084
Outside NYC	Orange	Total	82,092	72,316	4,404	639	1,176	12,272	23	8	135	52,006	225,071
Outside NYC	Orleans	Active	5,320	10,365	521	53	145	1,062	2	1	35	4,806	22,310
Outside NYC	Orleans	Inactive	528	757	63	9	27	121	0	0	2	586	2,093
Outside NYC	Orleans	Total	5,848	11,122	584	62	172	1,183	2	1	37	5,392	24,403
Outside NYC	Oswego	Active	17,037	31,121	1,643	143	340	3,685	6	3	39	14,213	68,230
Outside NYC	Oswego	Inactive	1,759	2,101	141	20	63	446	0	0	8	1,769	6,307
Outside NYC	Oswego	Total	18,796	33,222	1,784	163	403	4,131	6	3	47	15,982	74,537

NYSVoter Enrollment by County, Party Affiliation and Status
Voters Registered as of April 1, 2016

REGION	COUNTY	STATUS	DEM	REP	CON	GRE	WOR	IND	WEP	REF	OTH	BLANK	TOTAL
Outside NYC	Otsego	Active	10,386	12,864	517	139	141	2,100	5	2	36	7,081	33,271
Outside NYC	Otsego	Inactive	1,033	806	46	12	18	240	0	0	3	901	3,059
Outside NYC	Otsego	Total	11,419	13,670	563	151	159	2,340	5	2	39	7,982	36,330
Outside NYC	Putnam	Active	17,758	21,193	1,934	137	208	3,586	5	11	40	15,419	60,291
Outside NYC	Putnam	Inactive	1,474	1,486	140	12	13	318	0	0	4	1,329	4,776
Outside NYC	Putnam	Total	19,232	22,679	2,074	149	221	3,904	5	11	44	16,748	65,067
Outside NYC	Rensselaer	Active	28,135	23,588	4,256	363	1,109	7,538	13	10	35	28,303	93,350
Outside NYC	Rensselaer	Inactive	2,470	1,315	257	24	126	635	0	0	8	2,266	7,101
Outside NYC	Rensselaer	Total	30,605	24,903	4,513	387	1,235	8,173	13	10	43	30,569	100,451
Outside NYC	Rockland	Active	85,133	42,842	3,879	314	924	7,816	25	103	5	41,943	182,984
Outside NYC	Rockland	Inactive	5,660	2,549	181	38	57	613	2	1	0	3,039	12,140
Outside NYC	Rockland	Total	90,793	45,391	4,060	352	981	8,429	27	104	5	44,982	195,124
Outside NYC	Saratoga	Active	38,026	58,882	2,311	373	422	8,811	6	7	51	35,325	144,214
Outside NYC	Saratoga	Inactive	4,011	4,548	231	51	55	1,041	1	0	13	4,097	14,048
Outside NYC	Saratoga	Total	42,037	63,430	2,542	424	477	9,852	7	7	64	39,422	158,262
Outside NYC	Schenectady	Active	34,571	23,245	3,261	258	586	5,358	19	1	67	21,935	89,301
Outside NYC	Schenectady	Inactive	3,612	1,653	189	34	95	540	0	0	11	2,374	8,508
Outside NYC	Schenectady	Total	38,183	24,898	3,450	292	681	5,898	19	1	78	24,309	97,809
Outside NYC	Schoharie	Active	4,733	7,040	538	62	82	1,202	2	0	15	4,232	17,906
Outside NYC	Schoharie	Inactive	609	714	44	14	20	205	0	0	2	654	2,262
Outside NYC	Schoharie	Total	5,342	7,754	582	76	102	1,407	2	0	17	4,886	20,168

NYSVoter Enrollment by County, Party Affiliation and Status
Voters Registered as of April 1, 2016

REGION	COUNTY	STATUS	DEM	REP	CON	GRE	WOR	IND	WEP	REF	OTH	BLANK	TOTAL
Outside NYC	Schuyler	Active	3,235	4,644	247	62	69	717	1	0	0	2,550	11,525
Outside NYC	Schuyler	Inactive	266	303	16	5	12	88	0	0	0	277	967
Outside NYC	Schuyler	Total	3,501	4,947	263	67	81	805	1	0	0	2,827	12,492
Outside NYC	Seneca	Active	5,934	7,411	432	61	112	969	0	0	1	3,974	18,894
Outside NYC	Seneca	Inactive	468	500	22	7	16	110	0	0	0	452	1,575
Outside NYC	Seneca	Total	6,402	7,911	454	68	128	1,079	0	0	1	4,426	20,469
Outside NYC	St.Lawrence	Active	21,406	19,790	894	146	267	3,187	5	0	46	11,743	57,484
Outside NYC	St.Lawrence	Inactive	1,621	1,189	49	28	37	323	0	0	2	1,372	4,621
Outside NYC	St.Lawrence	Total	23,027	20,979	943	174	304	3,510	5	0	48	13,115	62,105
Outside NYC	Steuben	Active	14,022	27,831	958	154	263	2,936	6	2	40	9,874	56,086
Outside NYC	Steuben	Inactive	1,255	1,697	59	27	41	355	1	0	3	1,289	4,727
Outside NYC	Steuben	Total	15,277	29,528	1,017	181	304	3,291	7	2	43	11,163	60,813
Outside NYC	Suffolk	Active	303,955	295,671	22,443	1,800	4,363	42,905	82	13	593	249,128	920,953
Outside NYC	Suffolk	Inactive	21,104	15,771	1,024	150	337	2,888	3	2	42	15,490	56,811
Outside NYC	Suffolk	Total	325,059	311,442	23,467	1,950	4,700	45,793	85	15	635	264,618	977,764
Outside NYC	Sullivan	Active	16,865	12,235	1,055	141	230	2,303	10	1	33	10,933	43,806
Outside NYC	Sullivan	Inactive	3,316	1,516	122	26	54	417	0	0	2	2,009	7,462
Outside NYC	Sullivan	Total	20,181	13,751	1,177	167	284	2,720	10	1	35	12,942	51,268
Outside NYC	Tioga	Active	7,984	13,514	443	91	121	1,680	1	0	38	6,196	30,068
Outside NYC	Tioga	Inactive	658	875	25	12	21	169	0	0	4	657	2,421
Outside NYC	Tioga	Total	8,642	14,389	468	103	142	1,849	1	0	42	6,853	32,489

NYSVoter Enrollment by County, Party Affiliation and Status
Voters Registered as of April 1, 2016

REGION	COUNTY	STATUS	DEM	REP	CON	GRE	WOR	IND	WEP	REF	OTH	BLANK	TOTAL
Outside NYC	Tompkins	Active	25,749	11,623	394	456	217	2,240	8	0	70	10,730	51,487
Outside NYC	Tompkins	Inactive	3,768	1,290	48	71	44	400	0	0	12	2,461	8,094
Outside NYC	Tompkins	Total	29,517	12,913	442	527	261	2,640	8	0	82	13,191	59,581
Outside NYC	Ulster	Active	39,769	27,580	2,598	641	621	5,762	12	1	91	33,550	110,625
Outside NYC	Ulster	Inactive	3,820	1,934	165	75	74	544	1	0	17	3,321	9,951
Outside NYC	Ulster	Total	43,589	29,514	2,763	716	695	6,306	13	1	108	36,871	120,576
Outside NYC	Warren	Active	10,190	18,730	629	175	131	2,527	3	3	2	8,280	40,670
Outside NYC	Warren	Inactive	1,066	1,319	45	24	22	288	0	0	2	1,074	3,840
Outside NYC	Warren	Total	11,256	20,049	674	199	153	2,815	3	3	4	9,354	44,510
Outside NYC	Washington	Active	8,631	14,555	671	122	189	2,204	2	1	1	7,321	33,697
Outside NYC	Washington	Inactive	692	819	44	11	30	253	0	0	0	813	2,662
Outside NYC	Washington	Total	9,323	15,374	715	133	219	2,457	2	1	1	8,134	36,359
Outside NYC	Wayne	Active	13,057	21,700	1,518	131	269	2,782	3	1	61	13,502	53,024
Outside NYC	Wayne	Inactive	1,011	1,058	73	16	32	262	0	0	10	1,145	3,607
Outside NYC	Wayne	Total	14,068	22,758	1,591	147	301	3,044	3	1	71	14,647	56,631
Outside NYC	Westchester	Active	263,855	128,152	8,207	824	1,414	20,825	49	7	134	129,709	553,176
Outside NYC	Westchester	Inactive	26,561	12,200	690	92	197	2,354	0	0	15	14,297	56,406
Outside NYC	Westchester	Total	290,416	140,352	8,897	916	1,611	23,179	49	7	149	144,006	609,582
Outside NYC	Wyoming	Active	5,319	10,732	546	37	106	1,180	0	0	9	5,032	22,961
Outside NYC	Wyoming	Inactive	484	720	47	6	18	140	0	0	0	594	2,009
Outside NYC	Wyoming	Total	5,803	11,452	593	43	124	1,320	0	0	9	5,626	24,970

NYSVoter Enrollment by County, Party Affiliation and Status

Voters Registered as of April 1, 2016

REGION	COUNTY	STATUS	DEM	REP	CON	GRE	WOR	IND	WEP	REF	OTH	BLANK	TOTAL
Outside NYC	Yates	Active	3,190	6,360	240	49	60	664	3	2	5	2,666	13,239
Outside NYC	Yates	Inactive	239	313	16	3	9	60	0	0	1	290	931
Outside NYC	Yates	Total	3,429	6,673	256	52	69	724	3	2	6	2,956	14,170
Outside NYC Grand Tot		Active	2,510,740	2,140,518	131,558	16,921	29,192	331,288	672	298	4,064	1,558,004	6,723,255
Outside NYC Grand Tot		Inactive	210,598	131,117	8,024	2,004	3,439	30,890	12	4	585	141,757	528,430
Outside NYC Grand Tot		Total	2,721,338	2,271,635	139,582	18,925	32,631	362,178	684	302	4,649	1,699,761	7,251,685
Within NYC	Bronx	Active	491,052	37,190	2,855	549	2,634	12,489	136	14	87	85,653	632,659
Within NYC	Bronx	Inactive	74,076	5,952	440	82	524	2,197	9	0	21	15,103	98,404
Within NYC	Bronx	Total	565,128	43,142	3,295	631	3,158	14,686	145	14	108	100,756	731,063
Within NYC	Kings	Active	853,687	100,363	3,953	2,389	4,814	25,323	153	15	341	195,495	1,186,533
Within NYC	Kings	Inactive	91,961	10,290	443	341	693	3,533	0	0	46	24,743	132,050
Within NYC	Kings	Total	945,648	110,653	4,396	2,730	5,507	28,856	153	15	387	220,238	1,318,583
Within NYC	New York	Active	617,963	84,870	1,611	1,755	1,896	26,301	128	18	382	153,738	888,662
Within NYC	New York	Inactive	92,105	17,649	284	321	340	6,020	0	0	108	32,520	149,347
Within NYC	New York	Total	710,068	102,519	1,895	2,076	2,236	32,321	128	18	490	186,258	1,038,009
Within NYC	Queens	Active	675,661	115,703	5,228	1,426	3,329	25,672	143	14	221	205,778	1,033,175
Within NYC	Queens	Inactive	47,079	7,766	380	107	291	2,124	1	0	33	15,216	72,997
Within NYC	Queens	Total	722,740	123,469	5,608	1,533	3,620	27,796	144	14	254	220,994	1,106,172
Within NYC	Richmond	Active	119,328	76,352	4,359	354	1,082	9,099	29	14	91	53,756	264,464
Within NYC	Richmond	Inactive	8,247	3,918	220	22	110	630	0	0	7	3,712	16,866
Within NYC	Richmond	Total	127,575	80,270	4,579	376	1,192	9,729	29	14	98	57,468	281,330

NYSVoter Enrollment by County, Party Affiliation and Status
Voters Registered as of April 1, 2016

REGION	COUNTY	STATUS	DEM	REP	CON	GRE	WOR	IND	WEP	REF	OTH	BLANK	TOTAL
Within NYC Total		Active	2,757,691	414,478	18,006	6,473	13,755	98,884	589	75	1,122	694,420	4,005,493
Within NYC Total		Inactive	313,468	45,575	1,767	873	1,958	14,504	10	0	215	91,294	469,664
Within NYC Total		Total	3,071,159	460,053	19,773	7,346	15,713	113,388	599	75	1,337	785,714	4,475,157
Statewide Total		Active	5,268,431	2,554,996	149,564	23,394	42,947	430,172	1,261	373	5,186	2,252,424	10,728,748
Statewide Total		Inactive	524,066	176,692	9,791	2,877	5,397	45,394	22	4	800	233,051	998,094
Statewide Total		Total	5,792,497	2,731,688	159,355	26,271	48,344	475,566	1,283	377	5,986	2,485,475	11,726,842
